# Supplementary material for: Mitochondrial membrane potential regulates nuclear DNA methylation and gene expression through phospholipid remodeling
Source: bioRxiv. 2024 Jan 13:2024.01.12.575075. Preprint. [Version 1] doi: 10.1101/2024.01.12.575075 (PMC10802563; doi:10.1101/2024.01.12.575075)
Supplement: Supplement 1 [file media-1.pdf]

Figure S1

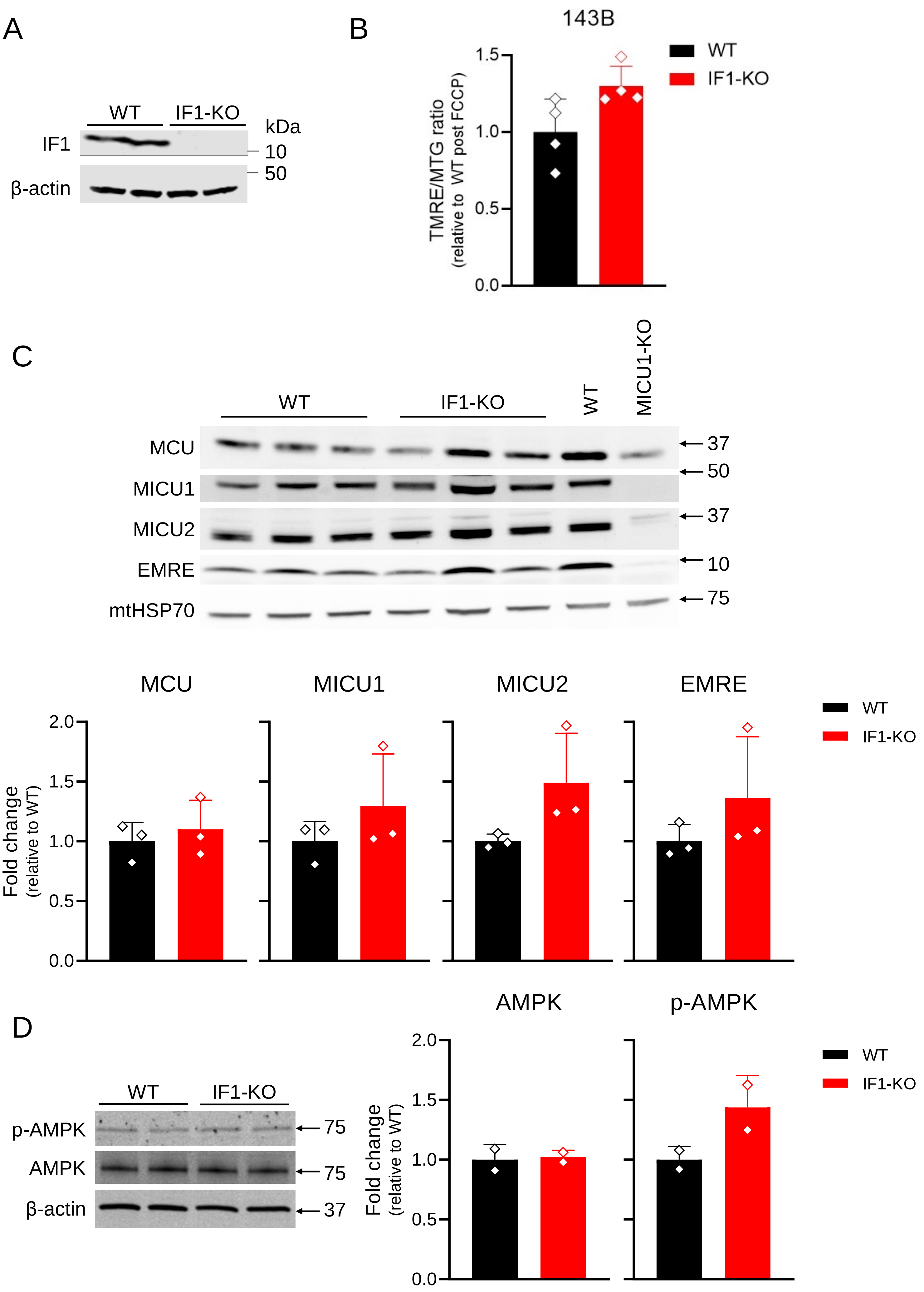

A
WT
IF1-KO
kDa
IF1
10
50
β-actin
B
143B
TMRE/MTG ratio
(relative to WT post FCCP)
1.5
1.0
0.5
0.0
WT
IF1-KO
C
WT
IF1-KO
WT
MICU1-KO
MCU
MICU1
MICU2
EMRE
mtHSP70
37
50
37
10
75
MCU
MICU1
MICU2
EMRE
WT
IF1-KO
Fold change
(relative to WT)
2.0
1.5
1.0
0.5
0.0
D
WT
IF1-KO
p-AMPK
AMPK
β-actin
75
75
37
AMPK
p-AMPK
Fold change
(relative to WT)
2.0
1.5
1.0
0.5
0.0
WT
IF1-KO

Figure S2

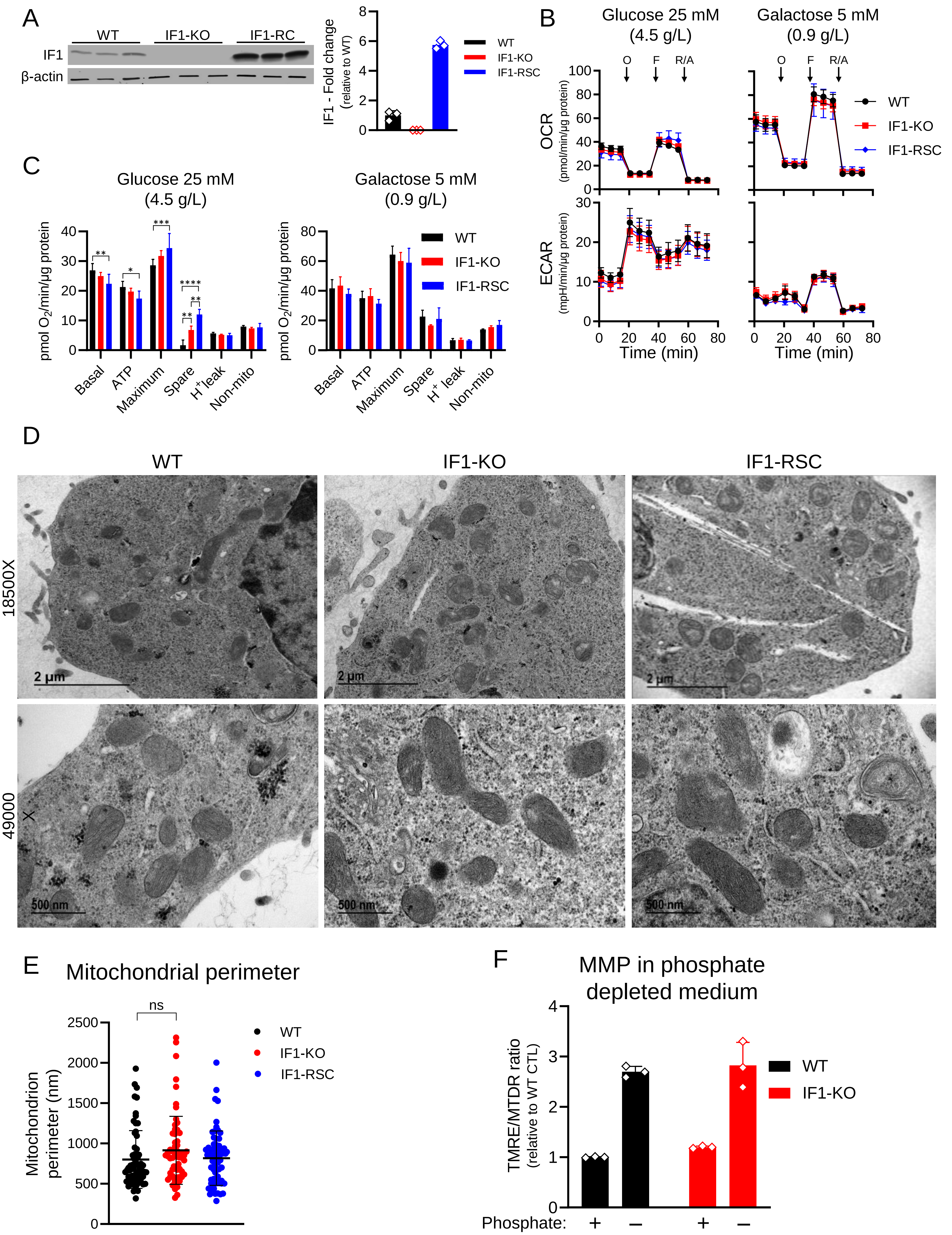
A
WT
IF1-KO
IF1-RC
IF1
β-actin
IF1 - Fold change (relative to WT)
WT
IF1-KO
IF1-RSC
B
Glucose 25 mM (4.5 g/L)
Galactose 5 mM (0.9 g/L)
O
F
R/A
OCR (pmol/min/µg protein)
ECAR (mpH/min/µg protein)
Time (min)
WT
IF1-KO
IF1-RSC
C
Glucose 25 mM (4.5 g/L)
Galactose 5 mM (0.9 g/L)
pmol O2/min/µg protein
Basal
ATP
Maximum
Spare
H+ leak
Non-mito
WT
IF1-KO
IF1-RSC
D
WT
IF1-KO
IF1-RSC
18500X
49000 X
2 µm
500 nm
E
Mitochondrial perimeter
Mitochondrion perimeter (nm)
ns
WT
IF1-KO
IF1-RSC
F
MMP in phosphate depleted medium
TMRE/MTDR ratio (relative to WT CTL)
WT
IF1-KO
Phosphate:
+
–
+
–

Figure S3

A

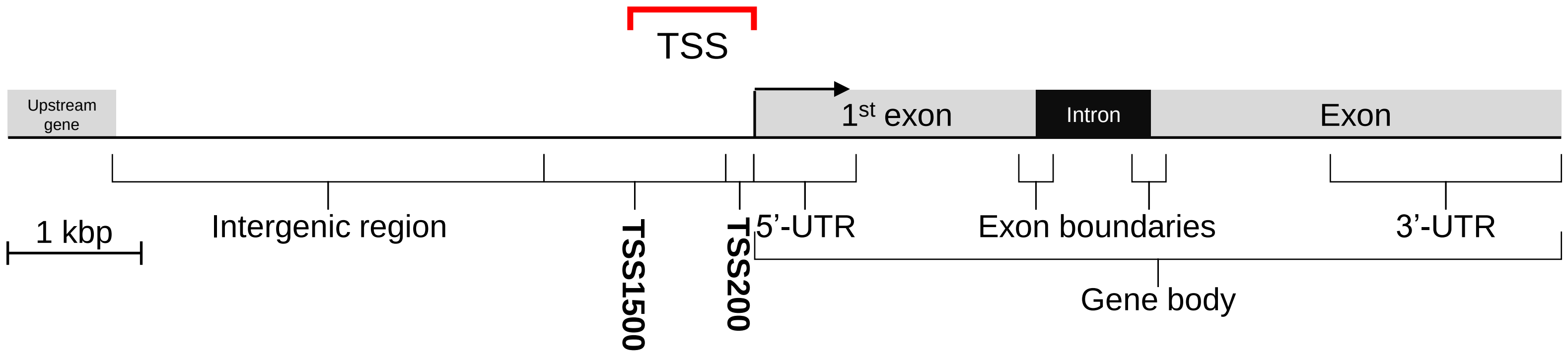

B

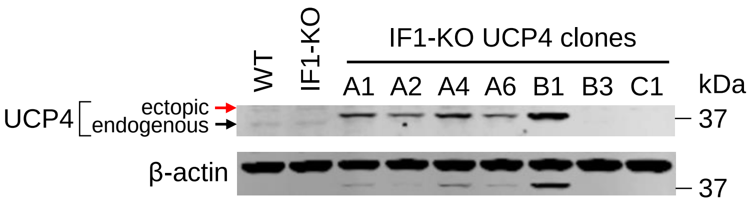

C

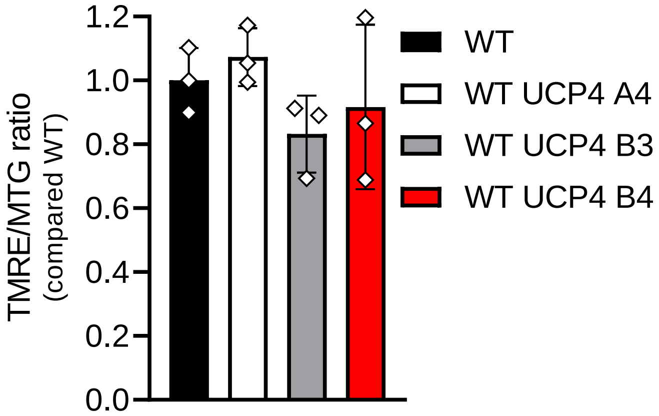

D

DNA methylation changes in RSC and UCP4 cells vs KO

	Rescued			UCP4		
Methylation	Hyper	Hypo	All	Hyper	Hypo	All
Full	1130	857	1987	1456	633	2089
Partial	2731	667	3398	1729	990	2719
No	4391	1816	6207	5161	1860	7021
Somewhat	3861	1524	5385	3185	1623	4808
Not full	7122	2483	9605	6890	2850	9740

Figure S4

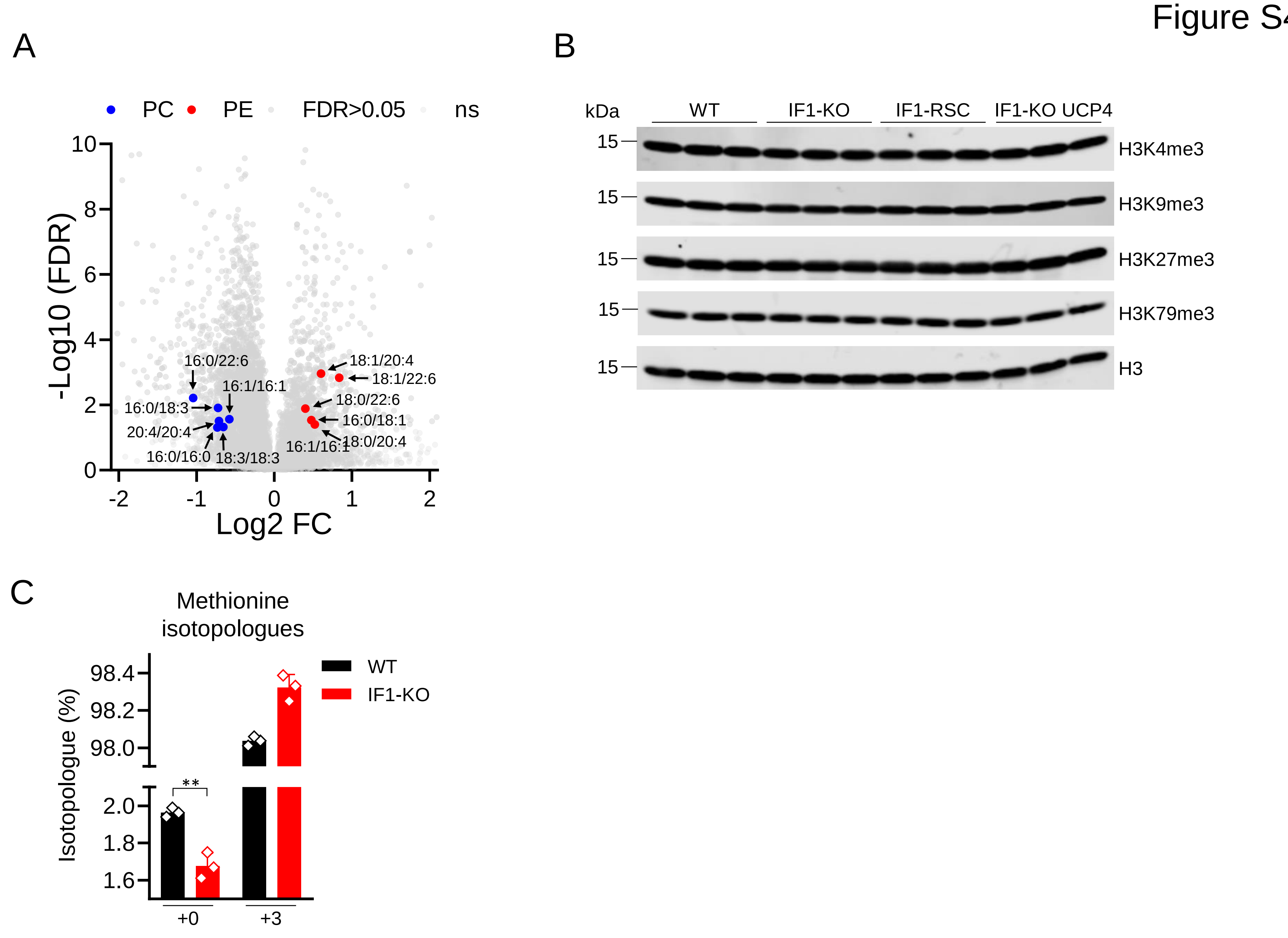

A
PC
PE
FDR>0.05
ns
-Log10 (FDR)
Log2 FC
16:0/22:6
16:1/16:1
16:0/18:3
20:4/20:4
16:0/16:0
18:3/18:3
18:1/20:4
18:1/22:6
18:0/22:6
16:0/18:1
16:1/16:1
18:0/20:4
B
kDa
WT
IF1-KO
IF1-RSC
IF1-KO UCP4
15
H3K4me3
H3K9me3
H3K27me3
H3K79me3
H3
C
Methionine isotopologues
Isotopologue (%)
WT
IF1-KO
**
+0
+3

Figure S5

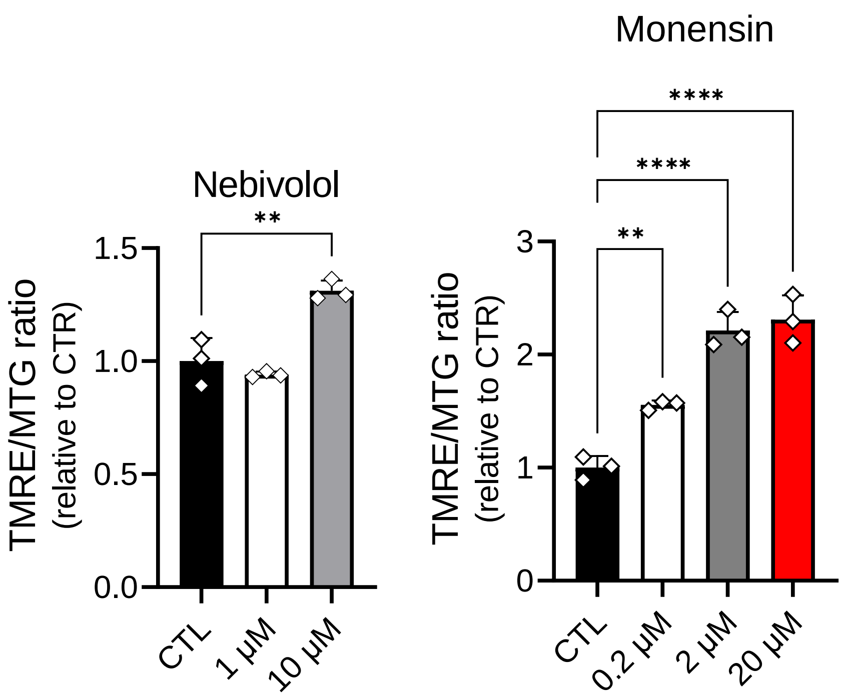

Nebivolol
Monensin
TMRE/MTG ratio
(relative to CTR)
**
****
****
**
1.5
1.0
0.5
0.0
3
2
1
0
CTL
1 µM
10 µM
CTL
0.2 µM
2 µM
20 µM
